# Mutation in West Nile Virus Structural Protein prM during Human Infection

**DOI:** 10.3201/eid2209.160132

**Published:** 2016-09

**Authors:** Yaniv Lustig, Robert S. Lanciotti, Musa Hindiyeh, Nathan Keller, Ron Milo, Shlomo Mayan, Ella Mendelson

**Affiliations:** Sheba Medical Center, Tel-Hashomer, Israel (Y. Lustig, M. Hindiyeh, N. Keller, E. Mendelson);; Centers for Disease Control and Prevention, Fort Collins, Colorado, USA (R.S. Lanciotti);; School of Public Health, Sackler Faculty of Medicine, Tel-Aviv University, Tel Aviv, Israel (M. Hindiyeh, E. Mendelson);; Faculty of Health Sciences, Ben-Gurion University of the Negev, Be'er sheva, Israel (R. Milo);; Barzilai Medical Center, Ashkelon, Israel (R. Milo, S. Mayan)

**Keywords:** West Nile virus, WNV, persistent infection, WNV mutation, prM protein, viruses, human infection

## Abstract

A mutation leading to substitution of a key amino acid in the prM protein of West Nile virus (WNV) occurred during persistent infection of an immunocompetent patient. WNV RNA persisted in the patient’s urine and serum in the presence of low-level neutralizing antibodies. This case demonstrates active replication of WNV during persistent infection.

West Nile virus (WNV) is a notable cause of neuroinvasive disease and febrile illness. In humans, WNV generates low viremia levels during infection (*1*). WNV is endemic in Israel and has been the cause of several disease outbreaks in recent years (*2*). Several subtypes of WNV lineage 1 have been phylogenetically identified in mosquitoes in Israel (*3*). However, no WNV sequence or isolation of viruses from humans in Israel had been reported since 2000 until the 2014 case we report here. In this study, we isolated and sequenced WNV lineage 1 and identified an amino acid mutation in the prM protein sequence that occurred between day 19 and day 28 of persistent viremia and viruria in a person with confirmed WNV encephalitis.

## The Study

A 56-year-old male gardener was admitted to Barzilai Medical Center in Ashkelon, Israel, on July 22, 2014, with a 7-day history of headache, abdominal pain, nausea, and fever (temperature 39°C); the date of hospital admission is designated as day 7 of his illness. His family recalled that he had received an unusually large mosquito bite 2 weeks before hospital admission. His medical history was remarkable for a thymoma B2 that was resected 3 years earlier without any evidence of myasthenia gravis. He did not receive immunosuppressive drugs or any other long-term drug therapy. 

On examination, he was drowsy, disoriented, and noncooperative. Marked neck rigidity was noted. Results of the remainder of the physical and neurologic examination were normal. A complete blood count revealed 13,750 leukocytes/μL, primarily neutrophils (88%). Follow-up blood counts, 10 and 50 days after admission, showed 6,400 and 6,500 leukocytes, of which 4,800 and 4,500 were neutrophils, respectively. Blood chemistry levels were within reference ranges, except for elevated blood glucose (131 mg/dL). Results of a computed tomography scan of his brain, without and with contrast media, were normal. Cerebrospinal fluid (CSF) examination revealed clear fluid containing 412 leukocytes/μL (285 neutrophils and 127 mononuclear cells/μL), a protein level of 234 mg/dL and glucose level of 55 mg/dL. Gram stain results were negative for bacteria. An electroencephalogram showed generalized slowing of brain electrical activity. Treatment with intravenous ceftriaxone and acyclovir (until herpes simplex virus infection was excluded by PCR) was initiated, and the patient’s mental status gradually improved. On the eighth day of hospitalization, pain developed in the left shoulder along with rapidly progressive weakness and atrophy in the left upper limb muscles over several days, mainly in the deltoid, supraspinatus, biceps, and triceps muscles. Treatment with antiinflammatory drugs and physiotherapy were initiated. Electrophysiologic studies 2 weeks later showed asymmetric denervation in the muscles innervated by C4–C7 nerve roots; the denervation was more prominent on the left side, compatible with an anterior-horn cell lesion. This segmental poliolike syndrome improved slowly and was still present a year later.

CSF and serum samples obtained on day 7 tested positive for specific WNV IgM and negative for WNV IgG (WNV IgM capture DxSelect and WNV IgG DxSelect; Focus Diagnostics Inc., Cypress, California, USA). Real-time reverse transcription PCR (RT-PCR) of CSF was negative for WNV RNA, but real-time RT-PCR of serum was positive for WNV RNA, confirming the diagnosis of WNV disease. Because RNA extracted from a urine sample obtained on day 12 was positive (8.8 × 10^7^ copies/mL) for WNV RNA, we monitored urine and serum samples for WNV RNA using real-time RT-PCR (*4*). The results demonstrated persistent viremia for 47 days and viruria for 61 days after illness onset (Table). In addition, infectious virus was isolated from 2 urine samples taken on days 12 and 15. We observed persistence of WNV RNA and virus isolation, despite the development of IgM and IgG, as well as WNV neutralizing antibodies (by ELISA and microneutralization) (Table).

**Table:** Serologic and molecular results in serum and urine samples from a patient with persistent WNV infection. Israel, 2014

Days after infection†	Days after illness onset‡	Serum, copies/mL	Urine, copies/mL	Serum IgM (ELISA result)§	Serum IgG (ELISA result)§	Serum neutralization titers‡	Amino acid in prM 20 (%)
14	7	6 × 10¶		Pos 1.8	Neg	Neg	T(100)
19	12		8.8 × 10^7^¶	ND	ND	ND	T (100)
22	15	2.5 × 10^4^	2.3 × 10^6^¶	Pos 8.07	Int 1.35	1:20	T (100)
23	16	3.6 × 10^3^	2 × 10^6^	Pos 8.16	Int 1.35	1:20	
24	17	8.3 × 10^3^	4.1 × 10^6^	Pos 8.12	Pos 1.65	ND	
25	18	1.2 × 10^4^	2 × 10^6^	Pos 8.29	Pos 1.98	1:20	
26	19	1.8 × 10^4^	1.8 × 10^6^¶	Pos 8.04	Pos 1.95	ND	T (100)
35	28	1 × 10^4^	1.3 × 10^5^ ¶	Pos 8.30	Pos 2.91	1:40	I (80), T (20)
50	43	6 × 10^2^	8.5 × 10^3^	Pos 8.20	Pos 3.27	1:40	
54	47	2 × 10^3^	6 × 10^2^	Pos 8.15	Pos 3.43	1:40	
61	54	Neg	1.1 × 10^4^	Pos 7.94	Pos 3.49	1:40	
68	61	Neg	6.8 × 10^1^	Pos 7.42	Pos 3.54	1:40	
76	69	Neg	Neg	Pos 7.35	Pos 3.61	1:40	

*I, isoleucine; Int, intermediate; ND, not done; Neg, negative; Pos, positive; T, threonine. †Based on patient’s memory of being bitten by a mosquito. ‡Days after start of headache, abdominal pain, nausea, and fever. §Results of ELISA IgM and IgG given in relative units. ¶Sample sequenced.

Whole-genome (96%) next-generation sequencing (Ion Torrent PGM system; Life Technologies, Grand Island, NY, USA) (*5*) of RNA extracted from the patient’s initial urine sample showed the highest identity with WNV sequences of the Mediterranean subtype within lineage 1, clade 1a, cluster 2 (Figure). Sequencing of the premembrane (prM), membrane, and envelope protein sequences (2,500 bp) obtained from the first serum sample showed identical sequences to those obtained from all subsequent urine samples taken until day 19. Sequencing of RNA from a urine sample obtained on day 28 revealed a single amino acid substitution, T20I (threonine to isoleucine), in WNV prM. Chromatogram results showed that this amino acid substitution was found in 80% of the sequences, displaying an almost complete change in virus sequence in <9 days. No other nucleotide mutations in the prM or the envelope proteins were identified.

**Figure:**
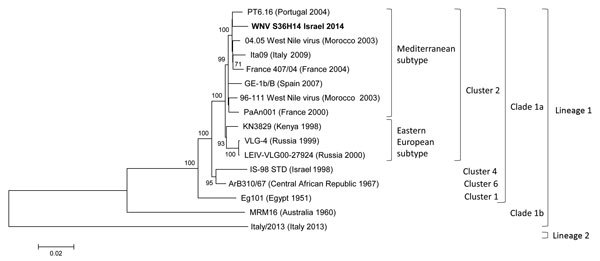
Phylogenetic analysis of West Nile virus (WNV) lineage 1 isolate from a patient with persistent WNV infection, Israel, 2014, compared with reference strains. The analysis was conducted on 96% of the WNV nucleotide sequence using the neighbor-joining method implemented in MEGA 6.0 software (http://www.megasoftware.net). The robustness of branching pattern was tested by 1,000 bootstrap replications. The percentage of successful bootstrap replicates is indicated at nodes, showing only values of >70%. A WNV lineage 2 sequence obtained from strain ITA 13 (GenBank no. KF647252) was used as an outgroup. Bold indicates the WNV lineage 1 strain sequenced in this study Scale bar indicates nucleotide substitutions per site.

## Conclusions

The phenomenon of WNV RNA persistence has been described in urine (*6*,*7*), plasma (*8*,*9*), and whole blood (*9*,*10*). However, the reason the virus persists in some patients but not in others is unknown. In this study, we followed the kinetics of viral clearance and antibody response of a patient with WNV persistence and demonstrated that, despite the development of IgM and IgG, substantial amounts of WNV RNA persisted in serum for 47 days and in urine for 61 days after illness onset. Notably, isolation of infectious virus from urine and appearance of an amino acid mutation in the prM of WNV on day 28 indicate not only persistence of WNV RNA but also active replication.

In assessing this case, we cannot exclude the possibility that the patient’s genetic background or underlying conditions may have played a role in the control of WNV infection and persistence. Although patients with thymoma B2 may, in rare cases, exhibit hypogammaglobulinemia and cellular immune dysfunction (*11*), this patient was considered immunocompetent because his medical history and follow-up after this infection did not show any indication of immune deficiency. The change in neutrophil count during acute infection is intriguing because neutrophils have been shown to serve as a reservoir for WNV replication during early infection and to contribute to viral clearance at a later stage of illness (*12*).

The amino acid mutation identified in this study (T20I prM) is conserved in all of the WNV strains sequenced so far. A previous study found that the mutation of T20 prM to aspartic acid (instead of the isoleucine identified here) affected glycosylation, heterodimer formation, and the secretion of WNV-like particles (*13*). This amino acid is located in the pr section of the prM protein, which is cleaved during virion maturation into a pr protein and a small membrane-anchored M peptide. Not all prM proteins are cleaved during egress, because virions containing at least some uncleaved prM protein are found in bulk virus populations and are infectious (*14*,*15*). The presence of prM on virions has been shown to increase the sensitivity of virus particles to neutralization by some envelope-specific antibodies (*15*). We observed only a small increase in neutralization between a sample containing the wild type virus (1:20, day 15) and a sample that mostly contained the mutated virus (1:40, day 28) (Table). These findings suggest that development of IgG was deficient. It is currently not possible to determine whether the mutation arose due to the persistent viremia and/or the antibody deficiency, triggered it, or contributed to the mutation.

Future studies should investigate whether T20I prM modifies the ability of WNV antibodies to interact with the virus and affect viral growth, modification, and infectivity. In addition, in light of the data described here, examining the dynamics of WNV clones and mutations in other cases of persistent infection would be worthwhile.
